# A Brief Study of Lachesis and Sabadilla

**Published:** 1887-05

**Authors:** C. Carleton Smith

**Affiliations:** Philadelphia


### A BRIEF STUDY OF LACHESIS AND SABADILLA.

C. Carleton Smith, M. D., Philadelphia.

It is within a comparatively short period of time that I became acquainted with the fact of the close similarity of the more special symptoms of the two drugs, Lachesis and Sabadilla.

Often have I been called upon to treat cases of chronic sore throat where the trouble recurred repeatedly, at short intervals, in all seasons; invariably locating itself in the left side, and spreading to the right; and yet which failed to be any more than temporarily benefited by Lachesis, though administered in all potencies. Having become acquainted with the close similarity existing between these remedies, I was at once struck by the fact that Sabadilla, as well as Lachesis, has as a leading symptom sore throat, beginning on the left side and spreading gradually to the right. Here, said I to myself, is, perhaps, the remedy for the Lachesis sore throat when it is met with in a chronic form, wherein Lachesis only acts palliatively in so many instances, or, in other words, improves, but does not hold the case. Following out this line of thought, and putting the drug to the test, I am thus far encouraged to believe that what, at this juncture, may perhaps be termed an inference, will very shortly be written down as an undeniable homœopathic fact—a fact which, in the hands of the members of the Lippe Club, I hope may receive, in due season, complete confirmation.

Let us now proceed to make a brief comparison of the more important symptoms of the throat and fauces of these two drugs.

*Lachesis*:—Uvula elongated, with feeling of lump in throat, which lump descends on swallowing, but returns at once.

*Sabadilla*:—Sensation of a skin hanging loosely in throat; obliged to swallow over it.

While swallowing, and when not swallowing, a feeling of a foreign body which he must swallow down, but can't.

*Lachesis*:—When swallowing fluids escape through the nose; worse, swallowing saliva—less from liquids, and even relieved by solids. Decided aggravation from hot drinks.

*Sabadilla*:—Stitches in throat only when swallowing, always going from left to right—as does also the suppuration. Continual desire to swallow, with deeply *cutting* pains, causing the whole body of the patient to *writhe*.

Aggravation, swallowing saliva, similar to Lach., but amelioration from swallowing warm food; the opposite of Lach.

Cannot swallow his saliva, must spit it out.

*Lachesis*:—Saliva abundant, tenacious, with bad odor from the mouth.

*Sabadilla*:—Saliva copious, and of a sweet taste.

*Lachesis*:—Tongue tastes sour, everything turns sour. Tongue catches behind the teeth when attempting to protrude it. Blisters on tip of tongue.

*Sabadilla*:—Taste, bitter or sweet, feels as if full of blisters. Cannot protrude tongue with sore throat.

*Lachesis*:—Breathing slow, difficult, whistling; chest feels constricted.

*Sabadilla*:—Sensation of narrowness of chest, with shortness of breath and wheezing.

*Lachesis*:—Cannot bear the least constriction of sore throat.

*Sabadilla*:—Sensation of constriction of throat and other parts of the body.

Under Lachesis complaints return regularly every fourteen days, but not at the same hour, while under Sabadilla they return every fourth day, precisely at the same hour.

The intolerable itching of the skin in Lachesis is relieved at once by plunging the parts in ice-cold water.

While the red spots and stripes of Sabadilla are more troublesome when exposed to the cold.

Lachesis has restless sleep.

Sabadilla, so drowsy he can scarcely overcome it.

